# What Is the Arrhythmic Substrate in Viral Myocarditis? Insights from Clinical and Animal Studies

**DOI:** 10.3389/fphys.2016.00308

**Published:** 2016-07-21

**Authors:** Gary Tse, Jie M. Yeo, Yin Wah Chan, Eric T. H. Lai Lai, Bryan P. Yan

**Affiliations:** ^1^Li Ka Shing Faculty of Medicine, School of Biomedical Sciences, University of Hong KongHong Kong, China; ^2^Department of Medicine and Therapeutics, The Chinese University of Hong KongHong Kong, China; ^3^Faculty of Medicine, Imperial College LondonLondon, UK; ^4^Department of Psychology, School of Biological Sciences, University of CambridgeCambridge, UK; ^5^Department of Epidemiology and Preventive Medicine, Monash UniversityMelbourne, VIC, Australia

**Keywords:** viral myocarditis, cardiac arrhythmia, mouse model, viral-induced cardiomyopathy, conduction, repolarization

## Abstract

Sudden cardiac death (SCD) remains an unsolved problem in the twenty-first century. It is often due to rapid onset, ventricular arrhythmias caused by a number of different clinical conditions. A proportion of SCD patients have identifiable diseases such as cardiomyopathies, but for others, the causes are unknown. Viral myocarditis is becoming increasingly recognized as a contributor to unexplained mortality, and is thought to be a major cause of SCD in the first two decades of life. Myocardial inflammation, ion channel dysfunction, electrophysiological, and structural remodeling may play important roles in generating life-threatening arrhythmias. The aim of this review article is to examine the electrophysiology of action potential conduction and repolarization and the mechanisms by which their derangements lead to triggered and reentrant arrhythmogenesis. By synthesizing experimental evidence from pre-clinical and clinical studies, a framework of how host (inflammation), and viral (altered cellular signaling) factors can induce ion electrophysiological and structural remodeling is illustrated. Current pharmacological options are mainly supportive, which may be accompanied by mechanical circulatory support. Heart transplantation is the only curative option in the worst case scenario. Future strategies for the management of viral myocarditis are discussed.

## Introduction

**Figure 1:**
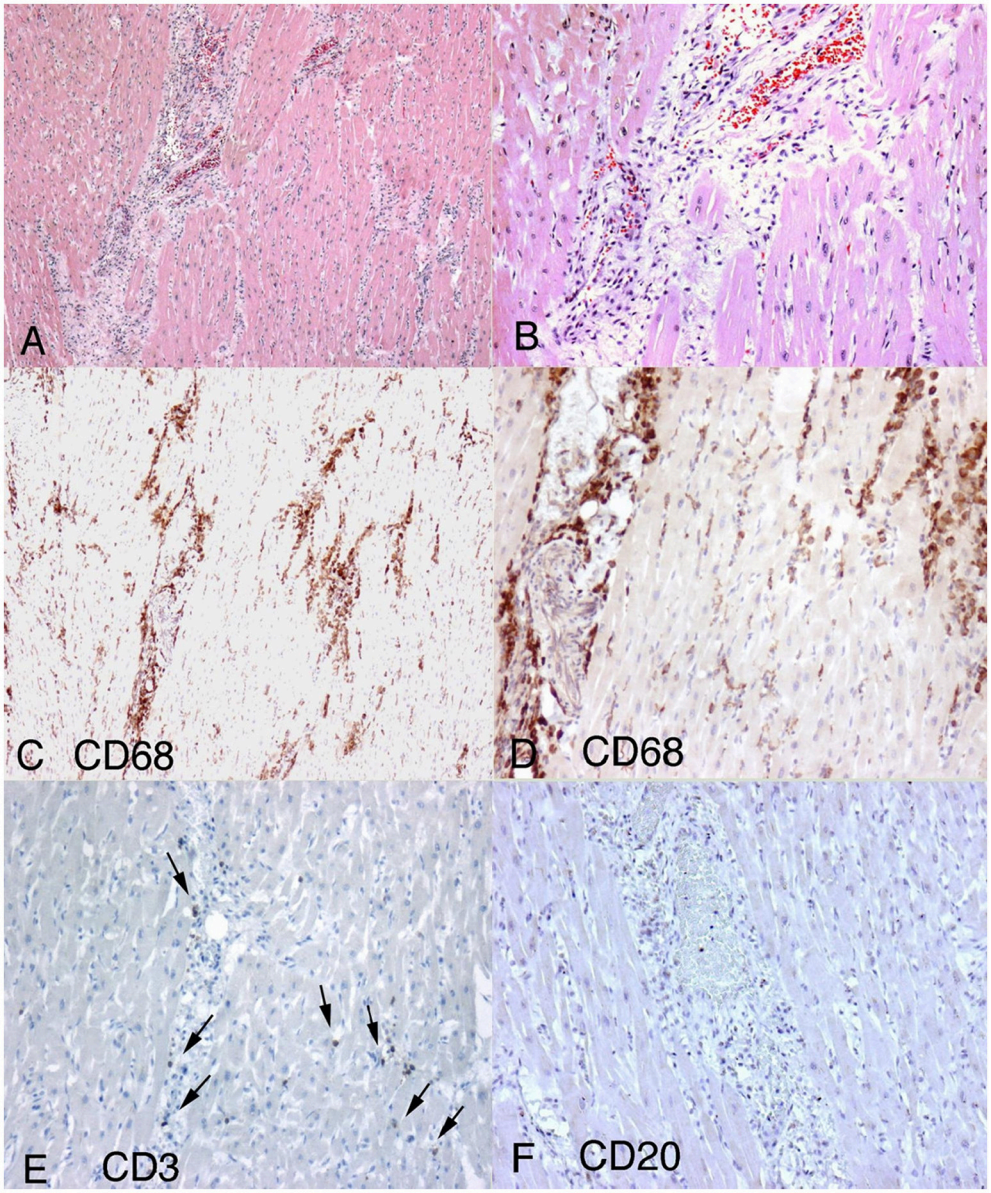
**Histopathological findings in parvoviral myocarditis**. **(A)** Diffuse interstitial myocardial inflammatory infiltrate more prominent around interstitial capillaries and composed of macrophages and lymphocytes (20×). **(B)** Hematoxilin-eosin stain showing vasocentric inflammation (40×). **(C,D)** CD68 positive macrophages were the most abundant cells present. (**C**-10X **D**-20X; **E)** Rare CD3 positive lymphocytes. **(F)** Essentially negative CD20 immunohistochemical stain. Figure and figure legends reproduced from Tavora et al. (2008) with permission.

**Figure 2:**
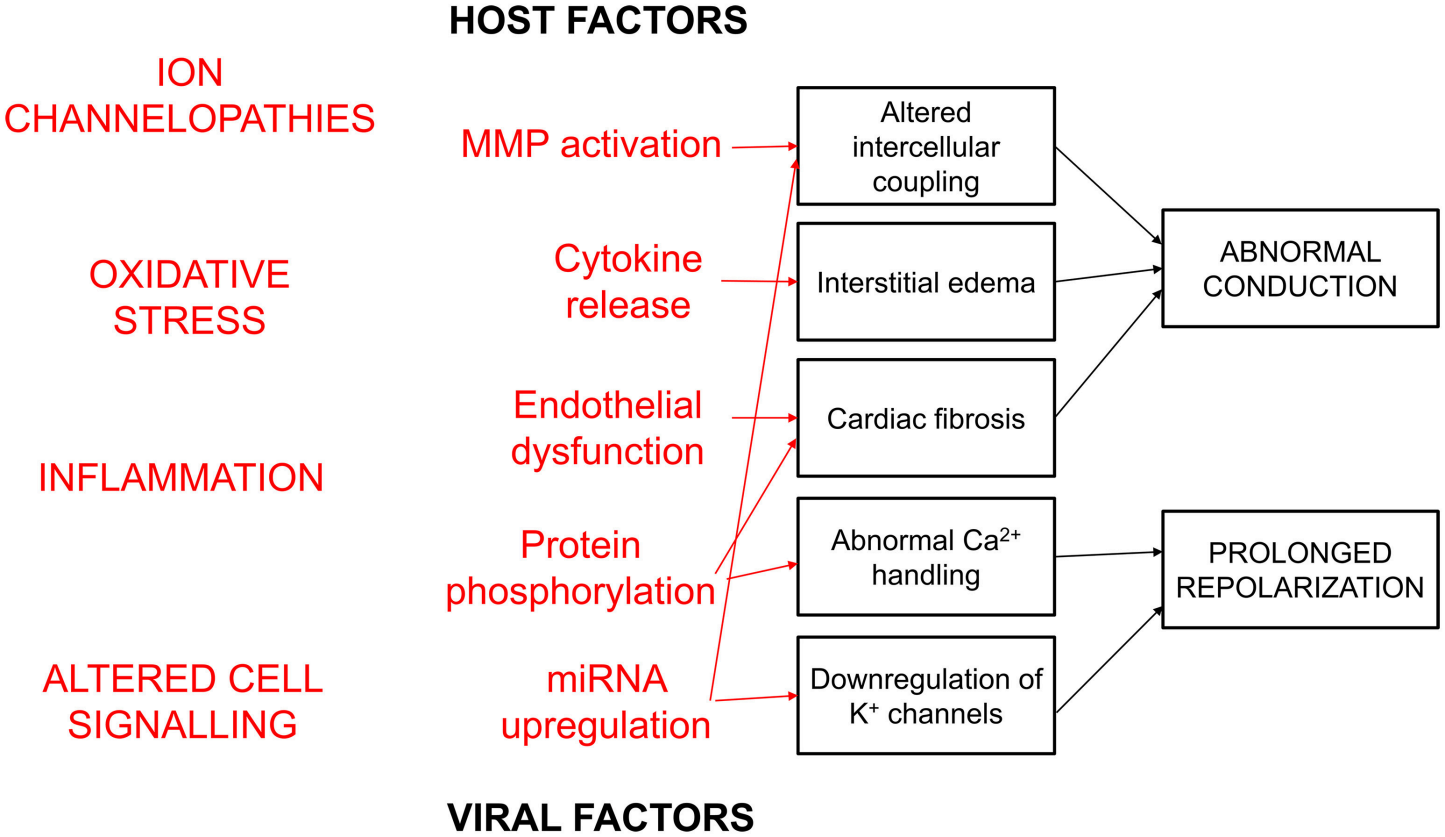
**Host and viral factors can induce structural and electrophysiological remodeling to induce cardiac arrhythmogenesis**. These include ion channelopathies, oxidative stress, inflammation, and altered intracellular signaling. Together, these act to alter intercellular coupling, produce interstitial oedema and fibrosis, which would lead to conduction abnormalities. Abnormal Ca^2+^ handling and K^+^ channel downregulation lead to abnormal repolarization.

Viral myocarditis is myocardial inflammation due to a viral infection. It is thought to be a major cause of sudden cardiac death (SCD) in the pediatric and adolescent population (Steinberger et al., 1996). Indeed, one study found that infants who suffered from SCD had mild fever and insomnia several days prior to their deaths, suggesting infection as a major contributor in this group (Gaaloul et al., 2016). At least 20 viruses have been implicated in myocarditis, but the commonest virus involved are Parvovirus B19 (PVB19), human herpes virus 6, adenovirus and coxsackievirus B3 (CVB3; Gaaloul et al., 2012). Table 1 summarizes the known virus strains, gene/protein targets, and estimated prevalence. Not all viral infections are the same: cardiotropic viruses are known to infect >90% of the human population, yet only 1–5% of these will develop viral myocarditis as proven histologically (Andreoletti et al., 2009). Patients can take a varied clinical course, from acute to chronic inflammation involving focal or diffuse areas of the myocardium (Fung et al., 2016). Figure 1 illustrates demonstrates the histology from a case of viral myocarditis due to PVB19, characterized by diffuse interstitial myocardial inflammatory infiltrate composed of CD68 positive macrophages, CD3 lymphocytes in an interstitial and perivascular distribution with minimal necrosis (Tavora et al., 2008). Some have insidious onset with limited inflammation, others undergo fulminant course with overwhelming inflammation or develop chronic heart failure from an autoimmune-mediated process (Heymans, 2006). There are some genetic predispositions, making some individuals more susceptible to viral myocarditis. For example, the commonest polymorphism for the KCNQ1 gene encoding for the slow inactivating K^+^ channel in Asians, appears to be protective against viral-induced arrhythmias (Steinke et al., 2013). Not all viruses are the same: some viruses such as CVB3 and adenovirus serotype 5 can induce more severe viral myocarditis (Savon et al., 2008; Valdes et al., 2008). For the post-mortem of infants suffering from SCD, only a minority of cases showed features of myocardial inflammation (Gaaloul et al., 2012), suggesting contributing factors, such as signals initiated by the viruses leading to ion channel dysfunction or electrophysiological and structural remodeling, to arrhythmogenesis. Fundamentally, viruses must have some means of subverting the host's machinery for their replication to ensure their own survival. This can be achieved by using the host's signaling mechanisms or the mircroRNA (miRNA) system to target the host's messenger RNAs for translational repression and degradation (Tomari and Zamore, 2005). The aim is to shut down the host's protein translation machinery and enhance viral pathogenicity or replication (Orom et al., 2008; Hemida et al., 2013; Tong et al., 2013; Ye et al., 2013). As we shall see later, altered cellular signaling, such as activation of kinases and enzymes, and upregulation of miRNAs, can lead to ion channel remodeling that can potentially reduce the threshold for arrhythmogenesis. Thus, the host's immune response or viral factor can induce electrophysiological or structural remodeling, resulting in action potential (AP) conduction or repolarization abnormalities to promote arrhythmogenesis (Figure 2; Tse and Yeo, 2015).

**Table 1:** **The prevalence of different viruses was obtained from Kühl et al. (2005) and Andreoletti et al. (2009)**.

**Virus**	**Type**	**Host target**	**Estimated prevalence**	**References**
Adenovirus	dsDNA	Common Coxsackievirus B-adenovirus receptor	8–23%	Bergelson et al., 1997; Bowles et al., 2003; Kühl et al., 2005; Andreoletti et al., 2009
Coxsackievirus	ssRNA	CD55, Common Coxsackievirus B-adenovirus receptor	2 to 50% (Up to 46% after transplantation)	Arbustini et al., 1992; Bergelson et al., 1997; Martino et al., 1998; Bowles et al., 2003; Andreoletti et al., 2009
Cytomegalovirus	dsDNA	Heparan Sulfate Proteoglycans, PDGFRα, EGFR, and integrin heterodimers	0.8–3%	Bowles et al., 2003; Chan et al., 2012
Echovirus	ssRNA	Human very late antigen 2 (VLA-2)	10.5%	Hughes et al., 2003; Kühl et al., 2005
Enterovirus	ssRNA	Enteroviral protease 2A directly cleaves dystrophin	8–32.6%	Badorff et al., 1999; Bowles et al., 2003; Kühl et al., 2005
Epstein-Barr virus	dsDNA	Increased latent membrane protein 1 is expressed in EBV latent cells	0–6%	Karjalainen et al., 1983; Bowles et al., 2003; Chimenti et al., 2004
Hepatitis B virus	dsDNA	Enters injured endothelium	<1%	Reis et al., 2007; Rong et al., 2007
Hepatitis C virus	ssRNA	CD68 (monocytes and macrophages)	2.9–3.8%	Matsumori et al., 2000; Reis et al., 2007; Matsumori, 2012
Herpes simplex virus	dsDNA		<1%	Bowles et al., 2003
Human herpes virus 6	dsDNA	?NK cells; infects endothelium	8–10.5%	Yoshikawa et al., 2001; Caruso et al., 2002; Kühl et al., 2005; Andreoletti et al., 2009
Human immunodeficiency virus 1 and 2	ssRNA	Gp120	Common in HIV positive patients	Shaboodien et al., 2013
Influenza virus	ssRNA	Ectopic trypsins	1.7–10% (up to 10% patients in influenza pandemics)	Bowles et al., 2003; Rezkalla and Kloner, 2010; Pan et al., 2011; Ukimura et al., 2012
Mumps virus	ssRNA		Up to 15% of mumps cases before introduction of vaccine (associated with endocardial fibroelastosis)	Rosenberg, 1945; Arita et al., 1981
Parvovirus B19	ssDNA	B19 receptor (erythrocyte P antigen)	1–36.6%	Porter et al., 1988; Bowles et al., 2003; Kühl et al., 2005; Andreoletti et al., 2009
Polio virus	ssRNA		Up to 40% of cases of poliomyelitis	Laake, 1951
Rabies virus	ssRNA	Invasion of neural tissue or blood cells	?	Ross and Armentrout, 1962; Cheetham et al., 1970; Venkat Raman et al., 1988; Liao et al., 2012
Respiratory syncytial virus	ssRNA	?	<1%	Huang et al., 1998; Bowles et al., 2003; Eisenhut, 2006
Rubella virus	ssRNA	?	?	Ainger et al., 1966; Kriseman, 1984
Vaccinia virus (smallpox vaccine)	dsDNA	?	<1–9.5%	Karjalainen et al., 1983; Casey et al., 2005
Varicella virus	dsDNA	?	?	Woolf et al., 1987; Rich and McErlean, 1993; Alter et al., 2001; Biocic et al., 2009; De et al., 2011

*? - Information not available*.

## Arrhythmogenesis can arise from AP conduction or repolarization abnormalities

**Figure 3:**
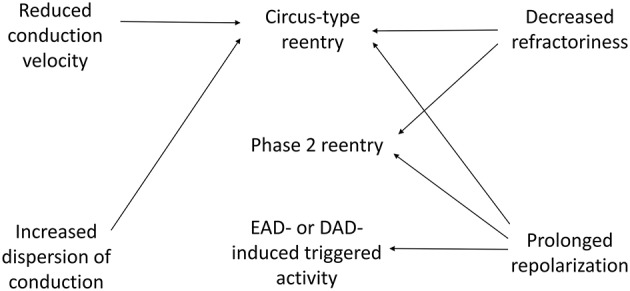
**Mechanisms of cardiac arrhythmias in viral myocarditis involves triggered activity and reentry**. Prolonged repolarization leads to development of early afterdepolarizations (EADs), whereas abnormal Ca^2+^ handling produces delayed afterdepolarizations (DADs). EADs and DADs can elicit triggered activity. Reduced conduction velocity (CV), increased CV dispersion and decreased refractoriness can increase the likelihood of circus-type reentry. Prolonged repolarization and decreased refractoriness can predispose to phase 2 reentry.

Mechanisms of arrhythmias can be divided into triggered activity and reentry (Figure 3; Tse, 2015; Tse et al., 2016m). Triggered activity arises from either early or delayed afterdepolarization phenomena (EADs and DADs), which are depolarization events occurring before the next AP. Normally the repolarization phase is determined by a balance of inward currents mediated by the Na^+^-Ca^2+^ exchanger (*I*_NCX_) and L-type Ca^2+^ channels (LTCC, *I*_Ca, L_), and outward currents mediated by a number of K^+^ channels (*I*_Kr_, *I*_Ks_, *I*_K1_,*I*_K, ATP_; Nerbonne, 2000; Tse et al., 2016j). Prolongation in action potential duration (APD) can result in LTCC reactivation, typically during phase 2 or phase 3 of the AP, leading to EADs (January et al., 1988). By contrast, DADs can develop under conditions of intracellular Ca^2+^ overload (Priori and Corr, 1990). This involves spontaneous release of Ca^2+^ from the sarcoplasmic reticulum via the ryanodine receptors (RyRs) and subsequent activation of the Na^+^-Ca^2+^ exchanger (NCX). Both EADs and DADs can therefore result in membrane depolarization, and if these are of sufficient amplitude, triggered activity can be elicited.

Reentry involves re-activation of the myocardium that has recovered from refractoriness, and may involve an obstacle for the circus-type, or without an obstacle in phase 2 reentry (Tse et al., 2016d). Circus-type reentry requires three conditions: reduced conduction velocity (CV) of the AP wave, so that the tissue ahead remains excitable, unidirectional conduction block to prevent APs traveling in opposite directions from extinguishing, and an obstacle (which can arise dynamically or be a fixed structural defect) around which the AP is able to circulate (Tse, 2015). Thus, a decrease in the wavelength of excitation (λ) given by the product of CV and reduced effective refractory period (ERP), would predispose to reentry (Smeets et al., 1986; Vaidya et al., 1999; Osadchii, 2010, 2014; Tse et al., 2012, 2016e,f,g,k,o).

Of these parameters, CV of the APs traveling through the myocardium, traditionally described by the core conductor theory, depends on both passive and active membrane properties (Tse and Yeo, 2015; Tse et al., 2016d). Passive properties refer to the biophysical parameters of axial resistance (r_i_), extracellular resistance (r_o_) and membrane capacitance (c_m_). The existence of electrical communication pathways between successive cardiomyocytes was shown, mediated by gap junctions (Spray and Burt, 1990). Six connexin (Cx) subunits make up a connexon and two connexons make up a gap junction. Since the discovery of gap junctions, it has been assumed that their electrical coupling is the primary mechanism by which cardiac conduction occurs. However, this is in conflict with experiments in heterozygous Cx43^+∕−−^ mice, which showed 45–50% reduction in Cx43 expression, but CV was either unaltered (Morley et al., 1999; Vaidya et al., 2001; van Rijen et al., 2004; Stein et al., 2009, 2011; George et al., 2015) or reduced by 23–44% (Guerrero et al., 1997; Thomas et al., 1998; Eloff et al., 2001). This suggests other mechanisms, such as ephaptic coupling, may have an important role in mediating cardiac conduction (Rhett and Gourdie, 2012; Lin and Keener, 2013, 2014; Rhett et al., 2013; Veeraraghavan et al., 2014a,b,c, 2015; George et al., 2015). This is clinically relevant because interstitial edema can increase extracellular volume, thereby reducing CV (Veeraraghavan et al., 2012).

Active properties refer to the voltage-gated conductance responsible for the AP upstroke, namely the Na^+^ channels. The effective refractory period (ERP) is the time over which Na^+^ channels are inactivated and cannot open again. They can be reactivated when the membrane potential is restored to the resting value. Thus, APD usually approximates ERP, i.e., a shorter repolarization time course usually leads to shorter ERP. When APDs are prolonged, sudden increase in heart rate can engage the steep portion of APD restitution curve, producing APD alternans, unidirectional conduction block, wave break and reentry (Hsieh et al., 2009, 2014, 2016; Tse et al., 2016m). Phase 2 reentry simply involves a difference in APD between two electrically connected regions, where conduction of the action potential dome from sites where it is maintained to sites where it is abolished can then result in an extrasystole (Shimizu et al., 2005). This is thought to underlie reentrant arrhythmogenesis in Brugada syndrome, and may be relevant in patients suffering from viral myocarditis with an unmasked Brugada phenotype.

## Host-mediated and viral-induced inflammation and can promote arrhythmogenesis by inducing ion channel abnormalities and cardiac remodeling

All of the above factors governing conduction or repolarization can be affected by myocardial inflammation or changes induced by the viruses to promote arrhythmogenesis. Thus, in a rat model of immune-mediated myocarditis, increased oxidative stress and inflammation can increase the release of inflammatory cytokines such as tumor necrosis factor-α and interleukin-6, leading to Ca^2+^/calmodulin Protein Kinase II (CaMKII) activation. This can phosphorylate the Ca^2+^ release channel, ryanodine receptor 2 (RyR2), to increase abnormal Ca^2+^ release from the sarcoplasmic reticulum (Tse et al., 2016n). Moreover, there is greater Ca^2+^ entry from the extracellular space (Tominaga et al., 1993). Both would lead to increased duration of Ca^2+^ transient, which would in turn prolong APD due to positive ${\text{Ca}}_{\text{i}}^{2+}$-APD coupling (Park et al., 2014a,b). This led to triggered activity, presumably via development of EADs, although DADs are also possible due to abnormal Ca^2+^ release. Regional differences in Ca^2+^ transients can also increase the heterogeneity in repolarization and produce arrhythmogenic APD alternans. In a rat model of autoimmune myocarditis, several ion channels mediating the fast transient outward (*I*_to, f_) and delayed rectifier (*I*_K__r_) currents were downregulated (Saito et al., 2002; Wakisaka et al., 2004). This led to prolongations of both ERP and APD, the latter being responsible for EADs and triggered activity. Similar reduction of repolarizing currents leading to APD prolongation has also been observed in mice with autoimmune myocarditis (Tang et al., 2007). Inflammation can also promote changes in the extracellular matrix (ECM). Thus, ECM composition is regulated by matrix metalloproteinases (MMPs), which are normally inhibited by tissue inhibitors of matrix metalloproteinases (TIMPs; Pauschinger et al., 2004). MMP activation during acute myocarditis can tip the balance toward ECM remodeling, in turn causing fibrosis. This would reduce CV by disrupting cardiomyocyte-cardiomyocyte coupling or increasing fibroblast-cardiomyocyte coupling, increasing r_i_ and C_m_, respectively (Tse and Yeo, 2015). Moreover, viral myocarditis predisposes to the development of dilated cardiomyopathy (DCM), which itself is arrhythmogenic. Interested readers are directed to this excellent article here for further discussion on the mechanisms by which myocardial infections by cardiotropic viruses lead to DCM and heart failure (Baksi et al., 2015).

Viruses can also alter the function or expression of ion channels or induce structural remodeling of the myocardium. CVB3 can increase the *I*_Ca_, leading to APD prolongation (Steinke et al., 2013). It also increases *I*_Kr_ and *I*_Ks_ initially but decreases them in the longer term, leading to APD shortening and prolongation, respectively. CVB3 can upregulate miR-1, which in turn disrupts cardiomyocyte-cardiomyocyte coupling by translational repression of the gene GJA1, which encodes for connexin-43 (Cx43; Xu et al., 2012). Together, these changes induced by CVB3 would produce Ca^2+^ overload and induce abnormalities in action potential repolarization and conduction, predisposing to both triggered activity and reentry. The cardiotropic PVB19 appears to target endothelial cells as opposed to cardiomyocytes (Bultmann et al., 2003). Since endothelial cells are found in the heart and can communicate with the adjacent cardiomyocytes, endothelial dysfunction may indeed be responsible for cardiac remodeling during inflammation. PVB19 a pro-apoptotic protein called viral protein NS1, which can activate caspase 3, leading to the degradation of the Na^+^/H^+^ exchanger (Lupescu et al., 2009). Its B19 minor capsid protein VP1 has intrinsic phospholipase A2 activity, which can increase the activity of Ca^2+^ release-activated Ca^2+^ channel (*I*_CRAC_), which is normally responsible for capacitative, store-operated Ca^2+^ entry by increasing *I*_Ca_ (Lupescu et al., 2006). PLA2 activity of VP1 is thought to underlie downregulation of Na^+^/K^+^-ATPase and a number of K^+^ channels (mediating *I*_Kr_ and inward rectifying currents, *I*_Kir_; Almilaji et al., 2013; Ahmed et al., 2014, 2015).

Finally, there may be interaction between genetic predisposition of ion channel dysfunction and viral myocarditis (Salerno et al., 2011; Juhasz et al., 2014). In a case series, patients who suffered from viral myocarditis complicated by ventricular fibrillation showed electrocardiographic features of Brugada, early repolarization and short QT syndromes (Salerno et al., 2011). Interestingly, not only were ventricular arrhythmias observed during the acute phase of the myocarditis but persistent ECG changes were observed after the inflammation has subsided, suggesting underlying abnormalities in ion channel function, predisposing to arrhythmogenesis during myocarditis. Indeed, as pointed out by these authors (Salerno et al., 2011), this could be due to temperature-dependent alterations in ion channel function (Pasquié, 2005). This notion is consistent with previous observations that infants suffering from SCD had mild fever before their deaths (Gaaloul et al., 2016), which would suggest fever as a trigger of the arrhythmia (Pasquié, 2005). This is also in keeping with previous associations between exacerbation of a Brugada pattern and a febrile state (Patane and Marte, 2010; Patane et al., 2010).

## Current management options and future therapy

Diagnosis of viral myocarditis can be difficult, and requires a series of investigations. Blood tests may reveal cardiac damage as reflected in raised troponins and high sensitive C-reactive protein assays (Guo, 2008). Polymerase chain reaction (PCR) can be used to detect viral nucleic acid materials for confirming a specific viral infection. Electrocardiography is non-specific, but can reveal conduction block, ST segment elevation or T wave abnormalities. Ventricular tachycardia or fibrillation may be observed. Echocardiography is used to determine ventricular function and rule out non-viral causes of heart failure, and can distinguish between acute from fulminant myocarditis (Felker et al., 2000). Cardiac magnetic resonance imaging is excellent for characterizing structural abnormalities, such as areas of fibrosis by late gadolinium enhancement (Vassiliou et al., 2014; Tse et al., 2015a,b) It is highly valuable in the diagnosis of myocarditis because it can detect interstitial edema during acute inflammation and fibrosis from a reparative process (Babu-Narayan et al., 2007; Petryka et al., 2014; Baksi et al., 2015). Interstitial edema reflects increased extracellular fluid volume, which would reduce CV by an ephaptic mechanism. Traditionally, the confirmatory test for diagnosing viral myocarditis was endomyocardial biopsy, which can be guided by electro-anatomical mapping to reduce the likelihood of false negatives. The criteria is a value more than 14 leukocytes/mm^2^ and a T-lymphocyte count of more than 7 cells/mm^2^ (Basso et al., 2013). However, due to advances in CMR technology, the use of biopsy is now limited when giant cell myocarditis is suspected.

For arrhythmic risk stratification, using different indices based on ECG parameters have been used for congenital arrhythmic syndromes and heart failure (Tse, 2016a,b,c; Tse and Yan, 2016a,b), but not for viral myocarditis. CMR can be used for stratifying patients into low and high risk group for developing ventricular arrhythmogenesis by quantifying the amount of interstitial edema and fibrosis, which would guide monitoring and therapy (Strauss and Wu, 2009; Mavrogeni et al., 2013; Baksi et al., 2015; Kallianos et al., 2015; Neilan et al., 2015; Sanguineti et al., 2015; Anzini et al., 2016).

The major problem of viral myocarditis is the limited number of drugs available for modifying the course of the disease and preventing the arrhythmic complications (Kindermann et al., 2012). The current treatment is supportive, using medications such as angiotensin converting enzyme inhibitors, beta blockers and spironolactone. Anti-arrhythmic agents are used when ventricular arrhythmias are observed. Mechanical circulatory support is potentially life-saving by allowing an interval for the return of heart pumping function or providing a bridge to heart transplantation, which may be required in the worst case scenario (Duncan et al., 2001). Other suggested approaches are immunosuppression, immunoglobulin, immunoadsorption, and anti-viral treatment (Jensen and Marchant, 2016). However, immunosuppressive therapy should be limited to giant cell myocarditis and lymphocytic myocarditis. The use of intravenous immunoglobulin is not recommended currently. There is a pressing need for drug development, and novel therapeutic agents that can reduce viral entry into cardiomyocytes, and viral-induced or host-mediate myocardial inflammation, which would reduce the arrhythmic burden in this patient population.

The use of animal models has advanced our understanding of the mechanisms of arrhythmias and provide a platform for assessing the efficacy of pharmacological therapy (Chen et al., 2016; Choy et al., 2016; Tse et al., 2016a,b,c,h,i,l). Thus, pre-clinical mouse studies have demonstrated the efficacy of Chinese medicinal extracts such as QiHong and Qishaowuwei formula in suppressing viral attachment and penetration, which significantly ameliorated CVB3-induced myocardium necrosis (Song et al., 2007; Fengqin et al., 2010). The benefits of traditional Chinese medicines in viral myocarditis thus warrant further investigation. Other novel therapies include mutation of the viral genome to induce the expression of cytokines, such as interferon-gamma, which can modulate the immune responses and prevent inflammation (Henke et al., 2008). Modulation of ion channel function may be useful for anti-arrhythmic therapy. Triggered activity can be suppressed by reversing APD prolongation and/or Ca^2+^ overload. Thus, EADs can be inhibited the late *I*_Na_ (Belardinelli et al., 2013), whereas DADs could be abolished by blocking RyR2 (Savio-Galimberti and Knollmann, 2015) or NCX (Sipido et al., 2006). K_ATP_ channel openers such as mexiletine, with previously demonstrated cardioprotective effects during ischaemia, could suppress APD prolongation during acute myocarditis and may therefor protect against Ca^2+^ overload, DADs and spatial heterogeneities in APDs (Niwano et al., 2012).

In conclusion, viral myocarditis is an important cause of mortality especially in infants, adolescents and young adults, predisposing to life-threatening cardiac arrhythmias. Current drug options are inadequate and are mainly supportive. More efforts need to be devoted to the development of novel pharmacological agents that can prevent viral invasion of cardiac tissue as well as viral- or host-induced inflammation, and reducing arrhythmic complications of the myocarditis.

## Author contributions

GT: Design of manuscript; drafted and critically revised the manuscript for important intellectual content; preparation of figures. JY: Drafted and critically revised the manuscript for important intellectual content. EL: Drafted and critically revised the manuscript for important intellectual content. BY: Interpreted primary research papers; critically revised the manuscript for important intellectual content.

### Conflict of interest statement

The authors declare that the research was conducted in the absence of any commercial or financial relationships that could be construed as a potential conflict of interest.
